# *Dipylidium caninum* Infection in Dogs and Humans in Bishoftu Town, Ethiopia

**DOI:** 10.3390/diseases9010001

**Published:** 2020-12-22

**Authors:** Fanta D. Gutema, Goitom W. Yohannes, Reta D. Abdi, Fufa Abuna, Dinka Ayana, Hika Waktole, Kebede Amenu, Adem Hiko, Getahun E. Agga

**Affiliations:** 1 College of Veterinary Medicine and Agriculture, Addis Ababa University, Bishoftu P.O. Box 34, Ethiopia; drfufex@gmail.com (F.A.); dinka_ayana@yahoo.com (D.A.); hikawaktole@yahoo.com (H.W.); kamenu@gmail.com (K.A.); 2 Humera Agricultural Research Center Humera, Tigray Agricultural Research Institute, Humera 6220, Ethiopia; yohansgoitom222@gmail.com; 3 Department of Veterinary Biomedical Sciences, College of Veterinary Medicine, Long Island University, Greenvale, New York, NY 11201, USA; reta.abdi@liu.edu; 4 College of Veterinary Medicine and Agriculture, Haramaya University, Haramaya P.O. Box 138, Ethiopia; adex.2010ph@gmail.com; 5 Food Animal Environmental Systems Research Unit, Agricultural Research Service, U.S. Department of Agriculture, Bowling Green, Kentucky, KY 40506, USA; getahun.agga@usda.gov

**Keywords:** *Dipylidium caninum*, prevalence, dogs, children, Bishoftu, Ethiopia

## Abstract

Dogs are reservoirs of many zoonotic diseases. In Ethiopia, the majority of owned dogs are semi-stray, freely roaming in the community. Studies reporting dog borne zoonotic diseases are scarce in Ethiopia. This study was conducted to assess *Dipylidium caninum* infection in dogs and in children with gastrointestinal complaints in Bishoftu Town, Oromia. We collected 384 fecal samples from dogs presented to veterinary teaching hospital and 259 stool samples from children presented to Bishoftu Hospital for clinical examination. Samples were first macroscopically examined for the presence of proglotids, followed by microscopic examination for the presence of eggs with the direct smear following flotation technique. The prevalence of *D. caninum* was 21% (95% CI: 16.6–24.9) in dogs. Although not statistically significant (*p* > 0.05), higher prevalence was detected in adult (11.9%), local breed (17.7%), and male (12.6%) dogs compared to young (8.59%), exotic breed (2.86%), and females (7.81%), respectively. *Dipylidium caninum* was detected in a stool sample obtained from a three year-old child (0.4%, 1/259). This study showed that the prevalence of *D. caninum* in the dogs is high while it is rare in children. Although the prevalence in children is negligible in this study, the high proportion of infected dogs can pose a significant risk of infection in the general human population. Public health risk can be reduced by eliminating the semi-roaming of owned dogs and proper management of dogs with regular deworming and prevention of environmental contamination with dog feces. Similarly, raising public awareness about dog borne zoonoses and avoiding contact with dog feces are important.

## 1. Introduction

Dogs have strong bond with humans, playing several roles in society such as guarding, hunting, and serving as pets in various countries [1]. For example, in the USA, about 85 million families own a pet and dogs are found in 63.4 million households [2]. Although dogs play important roles in the life of such families, they are also important reservoirs and sources of many zoonotic pathogens such as uropathogenic *Escherichia coli*, diarrhea causing agents (*Salmonella*, *Campylobacter*, and intestinal parasites), leptospirosis, brucellosis, Q fever, *Capnocytophaga canimorsus*, ringworm, *Cryptosporidium*, visceral larva migrans, and echinococcosis [3,4]. Dogs serve either as definitive or reservoir hosts for many zoonotic parasites posing major public health, economic, and social problems, particularly in developing countries where the movement and management of dogs are not commonly controlled [5,6,7].

*Dipylidium caninum* is a zoonotic parasite causing a disease named dipylidiasis in dogs and cats, and rarely in humans. It is commonly called dog tapeworm [8]. Fleas are the intermediate hosts while dogs are the final hosts in the developmental cycle of the tapeworm [9]. Humans acquire infection through fecal–oral transmission by incidental ingestion of infected flea containing *D. caninum* cycsticercoid [10]. Human infections are more likely to occur in young children who have an intimate contact with dogs and kiss or are licked by infected pet dogs [11]. Mild infections of *D. caninum* often are asymptomatic. However, it can cause abdominal pain, diarrhea, and anal pruritus in some individuals [12].

There are reported cases of dipylidiasis in humans from several countries and it is distributed worldwide [11,13,14,15]. Studies in Ethiopia showed high variability in the prevalence of *D. caninum* in dogs ranging from 6.6% [16] to 71% [17]. Information on the status of the disease in humans is lacking. In addition, dogs are the most neglected domestic animals and are usually never included in national research and development priority programs in Ethiopia. As a result, disease problems in dogs are rarely investigated and updated in the country. Dogs are owned by urban, rural, and pastoral households in Ethiopia where the dogs have a semi-stray type of free-roaming lifestyle with frequent contact with wildlife, livestock, the public, then finally return to their owner’s home [18,19]. Such unregulated free-roaming behavior can make owned dogs important spreaders of many zoonotic pathogens to their owner in Ethiopia [19,20,21]. Given the transmission potential of numerous zoonotic pathogens to humans through dogs [16], studies to better understand the epidemiology of dog borne zoonotic pathogens such as D. caninum in dogs and humans are important to design effective intervention measures. Therefore, the objective of the study was to assess the prevalence of *D. caninum* infection and risk factors in dogs and its occurrence in children in Bishoftu Town, Ethiopia.

## 2. Materials and Methods

### 2.1. Study Settings and Study Population

The study was conducted in Bishoftu Town, over a six month period from November 2017 to March 2018. Bishoftu is located in the central high land of Oromia, Ethiopia, 45 km southeast of Addis Ababa at 9°N latitude and 40°E longitude with an altitude of 1850 m above sea level. The area receives an average annual rainfall of 866 mm, 84% of which happens during the long rainy season (June to September). The dry season extends from October to February. The mean annual minimum and maximum temperatures are 14 °C and 26 °C, respectively with a mean relative humidity of 61.3%. According to the 2007 Ethiopian census report, the total human population of Bishoftu Town was estimated at 100,114 [22]. Bishoftu Hospital is a public hospital with a catchment population of approximately 1.2 million people. Its pediatrics department handles clinical cases of children under 14 years of age.

The study populations were all outpatient children at Bishoftu Hospital and dogs presented to a veterinary teaching hospital of the College of Veterinary Medicine and Agriculture of Addis Ababa University on the sampling days. Our inclusion criteria were gastrointestinal complaints such as anal pruritus, mild diarrhea, abdominal pain, and the presence of proglottids in the feces of dogs or in the stools of children that were collected for routine clinical examination and treatment. To determine the prevalence of *D. caninum* in dogs, fecal samples were consecutively collected from 384 dogs from all eligible dogs at the time of sampling occasions. The approximate age of the dogs was taken from the owners/attendants. For study involving humans, convenient stool samples from samples submitted to the laboratory for routine investigation during the study period were obtained from consecutive cases of 259 children with diarrhea. Both fecal and stool samples were examined grossly for the presence of proglottids. Samples were collected after obtaining written consent from the dog owners and the parents or guardians of the children. Both veterinary and medical hospitals were visited once per week to collect the samples.

### 2.2. Sample Collection and Processing

Approximately 10 g of feces samples were collected either from the rectum of physically restrained dogs or from the top layers of freshly voided feces. During sample collection, care was taken to ensure the welfare of dogs by gently restraining them in a facility built for this purpose. Samples were collected by a trained veterinarian. Samples were kept in universal plastic bottles, labeled, and transported to the parasitology laboratory in the College of Veterinary Medicine and Agriculture, Addis Ababa University. Clinical history, breed, age, and sex of dogs were recorded during sample collection. Stool samples (5–10 g) were collected using universal plastic bottles from the Bishoftu Hospital laboratory that were submitted for routine laboratory investigation and transported to the college on the same day by preserving in 10 mL of 10% formalin. Samples were processed on the same day or kept refrigerated at +4 °C until processed within 24 h of collection. All samples were examined first macroscopically for the presence of proglottids followed by microscopic examination for the presence of eggs using the flotation technique [23]. Magnesium sulfate with 1.2 specific gravity was used as a flotation fluid. Samples were considered positive when the proglottids and/or parasitic eggs/cysts with typical morphology and structure containing egg packets were observed [24].

### 2.3. Data Management and Analysis

Data were entered into a Microsoft Excel spreadsheet and analyzed using STATA software version 11.0 (StataCorp, College Station, TX, USA). Descriptive statistics such as frequency and percentage were used to describe the results. The prevalence of *D. caninum* was calculated by dividing the number of positive samples by the total number of samples examined. Logistic regression analysis was used to assess the association between the prevalence of *D. caninum* infection in dogs and putative risk factors such as sex, age, and breed of dogs. Confidence level was held at 95% and results were considered significant at *p* < 0.05.

## 3. Results

Among the 384 dogs examined, 79 (21%, 95% CI: 16.6–24.9) were positive for *D. caninum* infection. The prevalence was 12.8% in male, 11.9 in dogs older than one year and 17.7% in local breeds. Univariable logistic regression analysis showed no significant association between the prevalence of *D. caninum* infection and the risk factors considered (age, sex, and breed) (*p* > 0.05) (Table 1).

Of the 259 children examined, *D. caninum* was detected only in one child (0.4%). The child was a three year-old boy from Bishoftu town, with complaints of nausea, vomiting, and diarrhea for a duration of three days at the time of sampling.

## 4. Discussion

Many countries have reported the prevalence of multiple gastrointestinal parasites in community owned dogs, stray dogs, and dogs presented to veterinary health services. Most of these studies are based mainly on apparently healthy dogs as a main target of study population and rarely from clinical cases. Available information on the status of infection in humans is almost entirely from clinical case reports. The present study was a hospital-based study and reported the prevalence of *D. caninum* infection in dogs and children with gastrointestinal tract complaints presented for clinical examination to the veterinary teaching hospital and Bishoftu Hospital, respectively.

The 21% prevalence of *D. caninum* in dogs was comparable with 20% (95% CI: 12–29%) prevalence pooled from studies conducted in sub-Saharan Africa [25]. However, it was higher than the prevalence reported in dogs presented to veterinary hospitals, 8.2% (*n* = 98) in Nigeria [26] and lower than the 40% (*n* = 100) prevalence in Hidalgo county of Texas, USA [27]. Some community based studies have reported *D. caninum* infection in dogs in Ethiopia with a prevalence as low as 6.6% in Mekelle Town [16] and as high as 71% in Ambo Town [17]. A lower prevalence ranging from 0.1% (*n* = 3099) [28] to 0.8% (*n* = 2193) [29] was reported in dogs in Brazil, whereas a higher prevalence of up to 75% (*n* = 160) was reported in Nigeria [30]. Some recent studies have reported a prevalence of 4.5% (*n* = 200) in Nigeria [31], 1.9% (*n* = 263) in Spain [32], 16.5% (*n* = 103) in stray dogs in Mexico [33], 11.8% (*n* = 152) in Pakistan [34], 6% (*n* = 63) in Portugal [35], and 3.1% (*n* = 360) in stray dogs in Sudan [36]. The difference in the prevalence of *D. caninum* infection in dogs in different countries could be attributed to differences in the health care system and management of dogs, geographical areas, environmental sanitation, and the level of flea infestation in dogs [37,38,39]. In the present study, the semi-stray and free-roaming life style of dogs, which is common in Ethiopia, might have exposed dogs to *D. caninum* infection [18].

Age, sex, and breed of dogs were not associated with the prevalence of *D. caninum* infection in the present study. In agreement with this study, other studies have also reported lack of variations in the prevalence of *D. caninum* in different age groups, and between the sex of dogs [33,40,41]. Although there was no statistically significant association, a relatively higher frequency of *D. caninum* was observed in males, local breeds, and dogs older than one year. The observed high frequency in adult dogs could be due to the difference in frequency of exposure to flea infestation. Adult dogs, particularly stray dogs, are more likely to acquire *D. caninum* infection due to their behavior of traveling far away from home, which is common among dogs in Ethiopia that travel away from home, especially from villages to urban areas in search of food [16,39,42]. The higher frequency in male dogs compared to females might be due to the guarding stress and spending much more time in a confined house, which in turn can increase the chance of exposure to infection and can exacerbate re-infection as fleas can hide in the cracks [43]. Male dogs also usually move around in search of female dogs that could be on heat (estrus) and thereby scavenging bushes and waste that can increase their likelihood of exposure to *D*. *caninum* than female dogs [26].

In the present study, *D. caninum* was recovered from the stool sample of one child. Human infection with *D. caninum* is common among children. As reviewed by García-Agudo et al. [11], 16 cases of dipylidiasis were reported worldwide during a 20 year period up to 2014. These cases were reported from Europe, China, Japan, India, Sudan, Latin America, and the United States with 14 cases in children and the remaining two cases in adults. Recently, studies that reported dipylidiasis in humans from various countries are also available, for instance, in Greece, 10 cases of dipylidiasis in children with a mean age of 3.8 years, of which six of these were boys [13]. In Moscow, nine cases of dipylidiasis (eight in children and one in an adult) [14] were reported. Similar to the present study, a single case of *D. caninum* infection was reported from a 9-month old girl in Spain [11]; 17 month old boy in Henan Province, central China [15]; a 6 month old infant in the USA [44]; and a first-trimester pregnant woman in Ghana [45]. Our study reported the occurrence of *D. Caninum* in human stool sample for the first time from a three year old child in Ethiopia. Several human dipylidiasis cases have been reported from many countries with increasing trend [11]. The increasing reports from different geographical regions of the world signify its public health importance and should not be considered as a neglect disease any further.

The present study assessed only the occurrence of *D. caninum* in stool samples taken from outpatient children without investigating the potential risk factors. The study also did not assess any linkage between owning a dog or a cat at home and infections in children. Large scale community based studies are required to assess the magnitude of *D. caninum* in humans including adults and identify various potential risk factors such as owning pet dogs and cats, management of dogs and cats, and contact with dogs or their feces.

## 5. Conclusions

The present study showed a hospital-based prevalence of *D. caninum* infection of 21% in dogs and 0.4% in children. The observed high prevalence of *D. caninum* infection in dogs can be implicated as a high potential risk of transmission to humans and should be considered as a public health concern. Breaking the transmission cycle of *D. caninum* is needed by applying prevention and control measures such as periodic deworming, prevention of environmental contamination with dogs feces, controlling semi-stray and stray dogs, control of the intermediate host flea, and public education on dog borne zoonotic diseases and preventive measures.

## Figures and Tables

**Table 1:** Prevalence of *Dipylidium caninum* infection in dogs and its association with their demographic factors in Bishoftu Town, Ethiopia.

Variables		No Examined	No Positive	Prevalence (%)	OR (95% CI)	*p*-Value
**Sex**	Female	141	30	7.81	1	
	Male	243	49	12.76	0.98 (0.57–1.59)	0.868
**Age**	>1 year	247	46	11.98	1	
	≤1 year	137	33	8.59	1.38 (0.83–2.99)	0.206
**Breed**	Local	328	68	17.70	1	
	Exotic	56	11	2.86	0.98 (0.47–2.00)	0.955

OR: odds ratio; CI: confidence interval.
